# Alpha-Mangostin Improves Insulin Secretion and Protects INS-1 Cells from Streptozotocin-Induced Damage

**DOI:** 10.3390/ijms19051484

**Published:** 2018-05-16

**Authors:** Dahae Lee, Young-Mi Kim, Kiwon Jung, Young-Won Chin, Ki Sung Kang

**Affiliations:** 1 School of Pharmacy, Sungkyunkwan University, Suwon 16419, Korea; pjsldh@naver.com; 2 College of Pharmacy and Integrated Research Institute for Drug Development, Dongguk University-Seoul, Gyeonggi 10326, Korea; 0210121@hanmail.net; 3 Institute of Pharmaceutical Sciences, College of Pharmacy, CHA University, Sungnam 13844, Korea; pharmj@cha.ac.kr; 4 College of Korean Medicine, Gachon University, Seongnam 13120, Korea

**Keywords:** alpha mangostin, glucose response, insulin secretion, streptozotocin

## Abstract

Alpha (α)-mangostin, a yellow crystalline powder with a xanthone core structure, is isolated from mangosteen (*Garcinia mangostana*), which is a tropical fruit of great nutritional value. The aim of the present study was to investigate the anti-diabetic effects of α-mangostin and to elucidate the molecular mechanisms underlying its effect on pancreatic beta (β)-cell dysfunction. To assess the effects of α-mangostin on insulin production, rat pancreatic INS-1 cells were treated with non-toxic doses of α-mangostin (1–10 μM) and its impact on insulin signaling was examined by Western blotting. In addition, the protective effect of α-mangostin against pancreatic β-cell apoptosis was verified by using the β-cell toxin streptozotocin (STZ). Our results showed that α-mangostin stimulated insulin secretion in INS-1 cells by activating insulin receptor (IR) and pancreatic and duodenal homeobox 1 (Pdx1) followed by phosphorylation of phospho-phosphatidylinositol-3 kinase (PI3K), Akt, and extracellular signal regulated kinase (ERK) signaling cascades, whereas it inhibited the phosphorylation of insulin receptor substrate (IRS-1) (Ser1101). Moreover, α-mangostin was found to restore the STZ-induced decrease in INS-1 cell viability in a dose-dependent manner. In addition, treatment of INS-1 cells with 50 μM STZ resulted in an increase in intracellular reactive oxygen species (ROS) levels, which was represented by the fluorescence intensity of 2′,7′-dichlorodihydrofluorescein diacetate (DCFH-DA). This oxidative stress was decreased by co-treatment with 5 μM α-mangostin. Similarly, marked increases in the phosphorylation of P38, c-Jun N-terminal kinase (JNK), and cleavage of caspase-3 by STZ were decreased significantly by co-treatment with 5 μM α-mangostin. These results suggest that α-mangostin is capable of improving insulin secretion in pancreatic β-cells and protecting cells from apoptotic damage.

## 1. Introduction

Diabetes is a chronic metabolic disorder whose prevalence is increasing annually. It represents a major social issue associated with modern lifestyle worldwide [1,2,3]. Thus, the identification of compounds capable of preventing or treating diabetes is of great importance. Type 1 diabetes is an autoimmune disorder caused by pancreatic β-cells loss, while type 2 diabetes is a heterogeneous disease which is caused by pancreatic β-cells dysfunction and insufficient insulin release in response to glucose in peripheral tissues such as skeletal muscle (glucose disposal), liver (glucose production), and adipose tissue (lipolysis) [3,4,5,6,7]. The combination of failed metabolic response in skeletal muscle and pancreatic β-cells dysfunction causes an increase of glucose production by the liver [8].

Insulin resistance may be defined as a condition where a normal or elevated insulin concentration produces a failed metabolic response [4,9]. Treatment with high concentrations for a long time induces glucotoxicity in the cultured pancreatic β-cells. In addition, chronic hyperglycemia decreases insulin secretion from pancreatic β-cells and increases insulin resistance [10,11].

Several previous studies have investigated the molecular mechanisms underlying hyperglycemia-induced pancreatic β-cell dysfunction. Their results showed that high glucose concentrations decrease the phosphorylation of insulin receptor substrate (IRS) and phosphatidylinositol-3-kinase (PI3K) signaling pathways and expression of pancreatic and duodenal homeobox-1 (Pdx-1) in INS-1 cells [12,13,14,15].

Recently, there has been an increase in publications focusing on plant-derived natural products with anti-diabetic potential [16,17,18]. Several plant-derived natural products with different chemical structures can modulate blood glucose levels through various mechanisms and can be used to treat diabetes. For example, pycnogenol, isolated from *P. pinaster* and fenugreek seeds (*Trigonella foenum-graecum* L., Fabaceae), inhibits intestinal glucosidase and activates glucolytic and gluconeogenic enzymes, thus restoring glucose homeostasis in diabetic rats [17]. Cinnamaldehyde and procyanidin, type-A polymers isolated from cinnamon, improve glucose uptake and glycogen synthesis [18].

Alpha (α)-mangostin is a tetra-oxygenated diprenylated xanthane isolated from the stem bark of mangosteen (*Garcinia mangostana*) [6,19,20]. It has been shown to have strong pharmacological effects such as antioxidant, anti-inflammatory, antifungal, anti-invasive, and antimetastatic activities in human skin cancer cell lines [21]. In addition, α-mangostin showed protective effects against contrast-induced apoptotic damage in pig kidney cell line, with minimal toxicity [22]. Regarding diabetes-related researches, α-mangostin was found to improve glucose uptake by 3T3-L1 adipocytes, exert antihyperglycemic activity, and improve insulin insensitivity in type 2 diabetic rats induced by streptozotocin (STZ) [2,6,19]. However, the anti-diabetic effects of α-mangostin and its underlying molecular mechanisms in hyperglycemia-induced pancreatic β-cell dysfunction have not been identified yet.

In the present study, we investigated the effects of α-mangostin on insulin secretion and protein expression of insulin signaling pathways in INS-1 cells, and its effects on STZ-induced damage in INS-1 cells to elucidate the mechanisms underlying this effect.

## 2. Results

### 2.1. Effect of α-Mangostin on Glucose-Stimulated Secretion of Insulin (GSIS) in Cells

**Figure 1:**
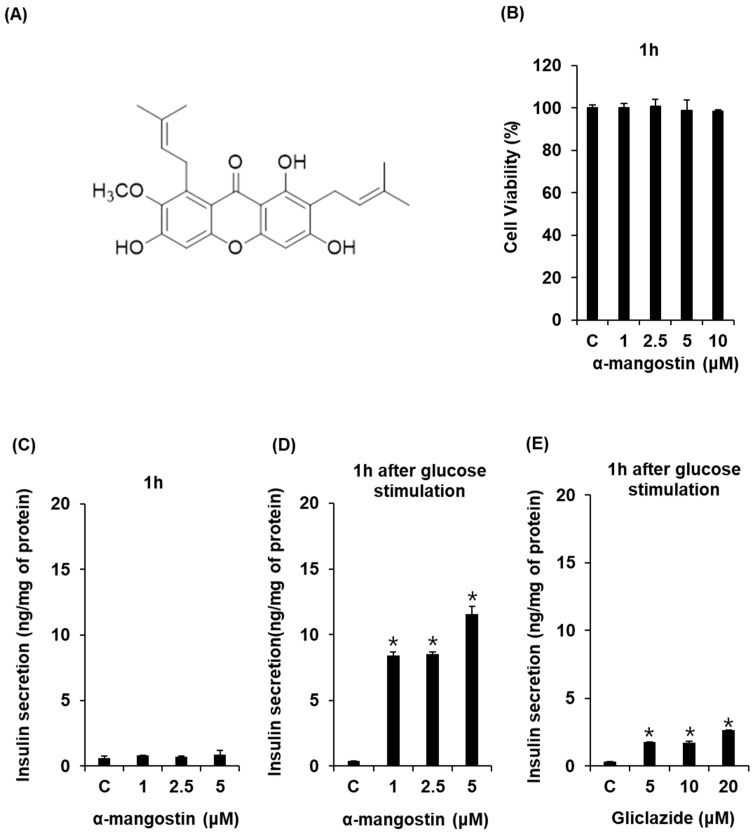
Effect of α-mangostin on glucose-stimulated insulin secretion (GSIS) in INS-1 cells. (**A**) Structure of α-mangostin. (**B**) Effect of α-mangostin on viability of INS-1 cells for 1 h. (**C**) Effect of α-mangostin on insulin secretion in INS-1 cells for 1 h in the absence of glucose. (**D**) Effect of α-mangostin on glucose-stimulated insulin secretion in INS-1 cells for 1 h. (**E**) Effect of gliclazide (positive control) on glucose-stimulated insulin secretion in INS-1 cells for 1 h. Insulin amount was normalized by total protein amount in the cell lysates. * *p* < 0.05 compared to the control value.

In order to determine the non-toxic dose ranges of α-mangostin, we assessed the cytotoxic effect of various concentrations of α-mangostin on INS-1 cells. As shown in Figure 1B, α-mangostin doses of 1, 2.5, 5, and 10 μM showed no toxic effects. In addition, α-mangostin at concentrations up to 5 μM has no effect on insulin secretion in the absence of glucose (Figure 1C), whereas it led to an increase in GSIS in a concentration-dependent manner (Figure 1D). The GSIS levels, calculated by glucose stimulation index values, were 27.6 ± 0.7, 32.8 ± 1.0, and 37.0 ± 0.5 for 1, 2.5, and 5 μM α-mangostin for 1 h, respectively (Figure 1D). This stimulatory effect of α-mangostin on insulin secretion was significantly higher than that of the positive control group (Figure 1E). These results indicate that α-mangostin stimulates insulin secretion in INS-1 cells for 1 h without inducing cytotoxicity.

### 2.2. Effect of α-Mangostin on Viability of INS-1 Cells for Various Time Points with High Glucose

**Figure 2:**
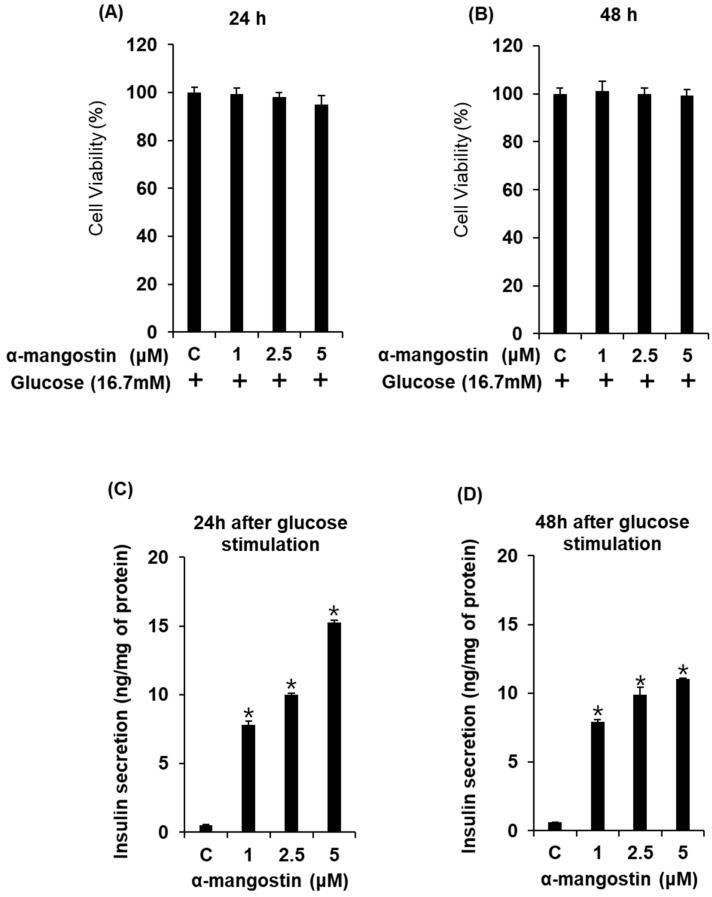
Effect of α-mangostin on viability and glucose-stimulated insulin secretion in INS-1 cells for various time points with high glucose. (**A**) Effect of α-mangostin on viability of INS-1 cells for 24 h. (**B**) Effect of α-mangostin on viability of INS-1 cells for 48 h. (**C**) Effect of α-mangostin on glucose-stimulated insulin secretion in INS-1 cells for 24 h. (**D**) Effect of α-mangostin on glucose-stimulated insulin secretion in INS-1 cells for 48 h. Insulin amount was normalized by total protein amount in the cell lysates. * *p* < 0.05 compared to the control value.

In order to investigate the chronic treatment of α-mangostin during glucotoxicity, we assessed the effect of α-mangostin on insulin secretion up to 48 h with high glucose concentration (16.7 mM) in INS-1 cells. As shown in Figure 2A,B, depending on time and concentration, there were no significant changes in cell viability by α-mangostin treatments. In addition, co-treatment with α-mangostin resulted in the concentration-dependent increase in glucose-stimulated insulin secretion (GSIS) up to 48 h (Figure 2C,D). These results indicate that the increase of GSIS by α-mangostin can be maintained up to 48 h.

### 2.3. Effect of α-Mangostin on the Protein Expression and Intracellular Ca^2+^ Levels Involved in Insulin Signaling in INS-1 Cells with Hyperglycemia-Induced Insulin Resistance

In order to investigate the underlying molecular mechanisms by which α-mangostin affects insulin secretion, Western blotting was performed to quantify the expressions of proteins in the insulin signaling pathways.

**Figure 3:**
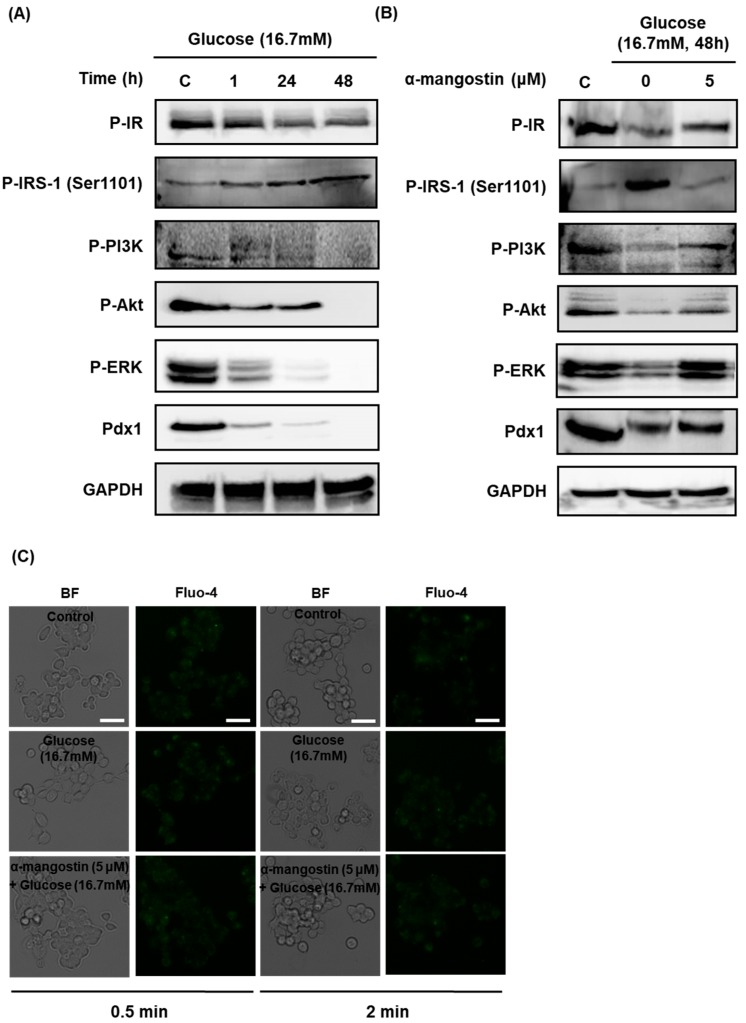
Effect of α-mangostin on the protein expression levels of P-IR, P-IRS-1 (Ser1101), P-PI3K, P-Akt, P-ERK, Pdx1 and intracellular Ca^2+^ in INS-1 cells with hyperglycemia-induced insulin resistance. (**A**) Protein expression levels of P-Insulin receptor, P-IRS-1 (Ser1101), P-PI3K, P-Akt, P-ERK, Pdx1, and GAPDH in INS-1 cells treated with 16.7 mM glucose (high glucose concentration) for different times. (**B**) Protein expression levels of P-Insulin receptor, P-IRS-1 (Ser1101), P-PI3K, P-Akt, P-ERK, Pdx1, and GAPDH in INS-1 cells treated or untreated with 16.7 mM glucose (high glucose concentration) and 5 μM α-mangostin for 1 h. (**C**) Intracellular Ca^2+^ in INS-1 cells treated with 16.7 mM glucose (high glucose concentration) and 5 μM α-mangostin for different times. Scale bar = 50 μm.

As shown in Figure 3A, the protein expression levels of phosphorylated insulin receptor (P-IR), P-PI3K, P-Akt, P-ERK, and Pdx1 were markedly decreased, while the protein expression of P-IRS-1 (Ser1101) was markedly increased in a time-dependent manner after treatment with high glucose concentration (16.7 mM).

The decreased expression of P-IR, P-PI3K, P-Akt, P-ERK, and Pdx1 protein treated with 16.7 mM glucose for 48 h was significantly recovered to the normal levels in the cells treated with 5 μM α-mangostin for 1 h. In addition, the elevated expression of P-IRS-1 (Ser1101) was reduced nearly to the normal level by co-treatment with α-mangostin (Figure 3B).

In order to investigate the involvement of second messengers by which α-mangostin affects insulin secretion, staining for intracellular Ca^2+^ was performed. However, there were no significant changes of intracellular Ca^2+^ in INS-1 cells treated with high glucose concentration (16.7 mM) and/or 5 μM α-mangostin for different times as shown in Figure 3C.

### 2.4. Effect of α-Mangostin on Streptozotocin (STZ)-Induced Damage in INS-1 Cells

**Figure 4:**
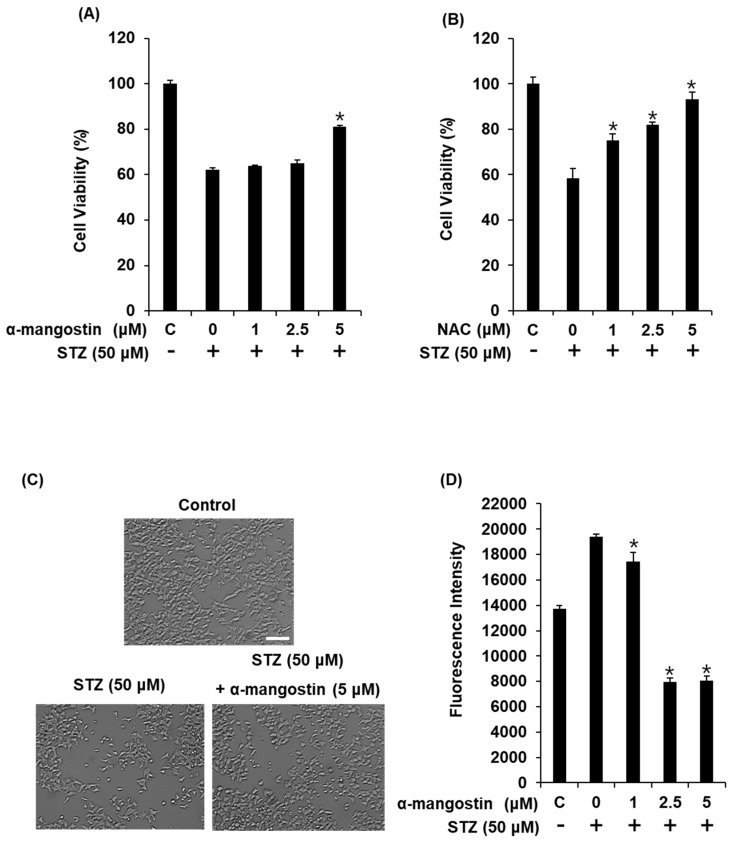
Effect of α-mangostin on STZ-induced damage in INS-1 cells. (**A**) Protective effect of α-mangostin against STZ-induced cell damage. (**B**) Protective effect of *N*-acetylcysteine (positive control) against STZ-induced cell damage. (**C**) Representative microscopic image showing the protective effects of α-mangostin on the cell morphology of INS-1 cells damaged by STZ. (**D**) Effect of α-mangostin on intracellular reactive oxygen species in INS-1 cells damaged by STZ. Scale bar = 50 μm. * *p* < 0.05 compared to the STZ-treated control value.

In our present study, the reduction in INS-1 cell viability induced by 50 μM STZ was restored by pre-treatment with α-mangostin in a concentration-dependent manner (Figure 4A). In Figure 4A, treatment with α-mangostin protected cells from death around 20% better than only STZ treated. This effect was similar to that of 2.5 μM *N*-acetylcysteine (positive control; Figure 4B). Co-treatment with 5 μM α-mangostin also resulted in significant improvements in the abnormal morphological changes related to apoptosis (cell shrinkage and membrane blebbing) observed in STZ-treated cells (Figure 4C). The levels of intracellular reactive oxygen species (ROS) in INS-1 cells are represented by the fluorescence intensity of 2′,7′-dichlorodihydrofluorescein diacetate (DCFH-DA). Treatment with 50 μM STZ resulted in a high fluorescence intensity, which was decreased by co-treatment with 5 μM α-mangostin (Figure 4D).

### 2.5. Effect of α-Mangostin on Mitogen-Activated Protein Kinases (MAPKs), PI3K/Akt, and Cleaved Caspase-3 in INS-1 Cells with STZ-Induced Damage

**Figure 5:**
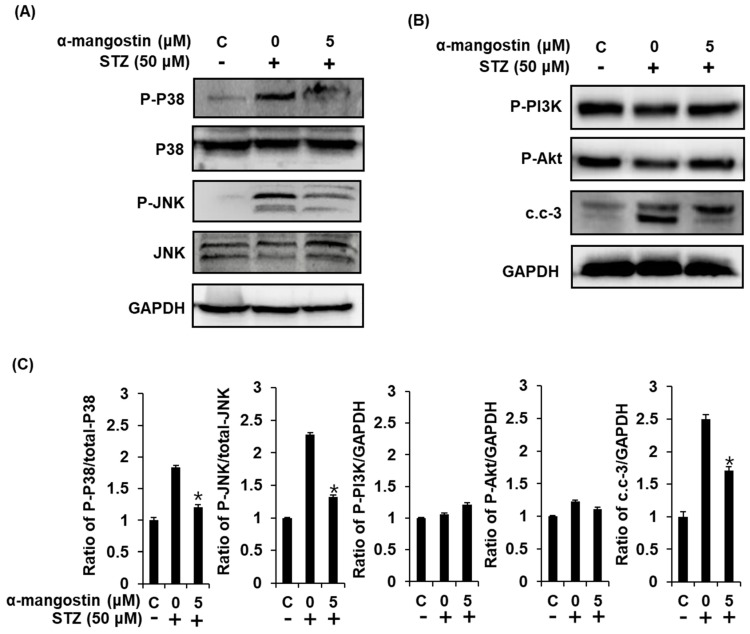
Effect of α-mangostin on MAPKs, PI3K/Akt, and cleaved caspase-3 (c.c-3) in INS-1 cells. (**A**) Protein expression levels of P-P38, P38, P-JNK, JNK, and GAPDH in INS-1 cells treated or untreated with STZ and 5 μM α-mangostin for 24 h. (**B**) Protein expression levels of P-PI3K, P-Akt, cleaved caspase-3, and GAPDH in INS-1 cells treated or untreated with STZ and 5 μM α-mangostin for 24 h. (**C**) Each bar graphs present the densitometric quantification results of Western blot bands. * *p* < 0.05 compared to the STZ-treated control value.

To further investigate the anti-apoptotic effects of α-mangostin, we evaluated the impact of 5 μM α-mangostin on the expression of P-P38, P38, P-JNK, JNK, P-PI3K, P-Akt, and cleaved caspase-3 proteins involved in STZ-induced apoptosis in INS-1 cells. Treatment with 50 μM STZ markedly increased the phosphorylation of P38 and JNK, and cleavage of caspase 3. However, these elevated protein expressions were significantly reversed by co-treatment with 5 μM α-mangostin (Figure 5). In addition, there were no significant changes of the expression of P-PI3K and P-Akt (Figure 5B).

Based on these results, STZ treatment induces the production of ROS, which play a critical role in phosphorylation of JNK, P38 MAPKs, and cleavage of caspase-3 leading to pancreatic β-cells apoptosis. We can conclude from this that the antioxidant activity of α-mangostin might be one of the main mechanisms underlying its protective effect against STZ-induced pancreatic β-cell damage.

## 3. Discussion

Insulin resistance and pancreatic β-cell dysfunction are the main hallmarks of type 2 diabetes [23,24]. Homeostasis deficiency eventually results in chronic hyperglycemia, which leads to glucose toxicity to organs and tissues, a decrease in insulin secretion, and an increase in insulin resistance in pancreatic β-cells [10,25].

In our study, α-mangostin stimulates insulin secretion in INS-1 cells for 1 h without inducing cytotoxicity. In addition, the protein expression levels of phosphorylated insulin receptor (P-IR), P-PI3K, P-Akt, P-ERK, and Pdx1 were markedly decreased, while the protein expression of P-IRS-1 (Ser1101) was markedly increased in a time-dependent manner after treatment with high glucose concentration (16.7 mM).

Cellular glucose uptake and metabolism are regulated by binding of insulin to the IR and activating its intrinsic tyrosine kinase activity. Phosphorylation of IR activates the IRS-1 and IRS-2, which are major substrates of IR that play a central key role in metabolic actions of insulin [26,27]. Above all, IRS-1 is a critical pathway linked to the IR that contains many potential serine phosphorylation sites [27]. The present study demonstrated that phosphorylation of IRS-1 at serine 1101 yields tyrosine-phosphorylated IRS-1, which interacts with p85 regulatory subunit of PI3K, leading to its activation. Activation of PI3K leads to stimulation of Akt, which plays a central key role in insulin resistance and pancreatic dysfunction thus impairing glucose uptake, glycogen synthesis, and protein synthesis [13,27]. It also regulates the expression and activity of a wide range of proteins such as enzymes, transcription factors, and proteins affecting pancreatic β-cell death and survival [28,29]. Akt then activates MAPK/ERK pathway, leading to cell proliferation or differentiation and regulation of gene expression [13]. It has kinase activity to pancreatic and Pdx1. Pdx1 is a transcription factor that regulates pancreatic β-cell growth, insulin synthesis, and gene expression [30,31]. In the present study, we observed that α-mangostin stimulated insulin secretion in INS-1 cells via activating IR and Pdx1 followed by PI3K, Akt, and ERK signaling cascades, whereas it inhibited the phosphorylation of IRS-1 (Ser1101). The previous studies showed that the increase in intracellular Ca^2+^ levels of the pancreatic β-cell surface stimulates insulin granule fusion with the plasma membrane, resulting in insulin exocytosis from pancreatic β-cells [32]. Thus, we tested whether intracellular Ca^2+^ levels increased time-dependent in INS-1 cells co-treated with high glucose concentration (16.7 mM) and α-mangostin (5 μM). Microscopic images revealed that the treatment with α-mangostin (5 μM) did not affect intracellular Ca^2+^ levels.

STZ, an antibiotic and anticancer agent, is the most commonly used agent for the experimental induction of diabetes in both cells and animal models [1,33,34]. It works by inducing oxidative stress in pancreatic β-cells and thus activating kinases known as stress-activated kinases such as JNK and p38 MAPKs pathways, which result in hyperglycemia, increased expression of ROS, osmotic stress, and release of pro-inflammatory cytokines [35,36]. In addition, oxidative stress can lead to pancreatic β-cell loss through apoptosis or necrosis. STZ also affects many cellular processes such as proliferation, differentiation, response to inflammation, and autophagy [3,37,38,39,40,41].

In our present study, the reduction in INS-1 cell viability induced by 50 μM STZ was restored by pre-treatment with α-mangostin in a concentration-dependent manner. The oxidative stress resulting from the reduction in INS-1 cell viability by 50 μM STZ was decreased by co-treatment with 5 μM α-mangostin. In addition, we evaluated that co-treatment with 5 μM α-mangostin effectively suppressed the pancreatic β-cell apoptosis induced by STZ through decrease of the phosphorylation of P38 and JNK.

Our results demonstrate that α-mangostin, an active component of mangosteen (*Garcinia mangostana*), not only enhances insulin production by activating insulin signaling, but also protects pancreatic β-cells against STZ-induced apoptotic damage. Therefore, it is possible to use α-mangostin for diabetes treatment for improving insulin resistance and function as well as suppression of pancreatic damage during elderly sepsis, where multi organ shock and pseudo-diabetes of type 1 happen. Our future study should be performed to identify physiological relevance in animal model, or ex vivo primary islets.

## 4. Materials and Methods

### 4.1. Chemicals

STZ and 2′,7′-dichlorodihydrofluorescein diacetate (DCF-DA) were purchased from Sigma Aldrich (Saint Louis, MO, USA). The cell viability assay kit (Ez-Cytox, Seoul, Korea) was purchased from the Daeil Lab Service Co. (Seoul, Korea). The rat insulin enzyme-linked immunosorbent assay (ELISA) kit (AKRIN-010T, Shibayagi Co. Ltd., Gunma, Japan) was purchased from Gentaur (Shibayagi Co., Ltd., Gunma, Shibukaw, Japan). RIPA buffer and bicinchoninic acid (BCA) protein assay kit were purchased from Cell Signaling (Danvers, MA, USA) and Thermo Scientific (Carlsbad, CA, USA), respectively. α-Mangostin was purified (>98.0% of purity) at the College of Pharmacy, Dongguk University (Seoul, Korea) (Figures S1–S4).

### 4.2. Cell Culture

The INS-1 rat insulinoma cell line was purchased from Biohermes (Shanghai, China). INS-1 cells were cultured in RPMI-1640 media (Cellgro, Manassas, VA, USA) supplemented with 1% penicillin/streptomycin (Invitrogen Co., Grand Island, NY, USA), 10% FBS, 11 mM d-glucose, 2 mM l-glutamine, 10 mM hydroxyethyl-piperazineethane-sulfonic acid (HEPES), 0.05 mM 2-mercaptoethanol, and 1 mM sodium pyruvate at 37 °C in a humidified atmosphere with 5% CO_2_.

### 4.3. Cell Viability Assay

Cell proliferation was evaluated using the Ez-Cytox cell viability detection kit as reported previously [42]. Cells were incubated with Ez-Cytox reagent (10 μL/well) for 2 h. Then, absorbance values for each well were measured at 450 nm using a PowerWave XS microplate reader (Bio-Tek Instruments, Winooski, VT, USA).

### 4.4. Reactive Oxygen Species (ROS) Measurement

Intracellular ROS generation was measured by using DCF-DA, which detects hydroxyl and peroxyl radicals as well as other species within the cell. The INS 1 cells were prepared for the experiment by seeding in a dark, clear-bottomed container at a density of 2 × 10^4^ cells/mL (100 μL/well), at 80% to 90% confluence, and incubated for 24 h to adhere in the 96-well cell culture plate. Cells were treated with different concentrations of α-mangostin (0, 1, 2.5, and 5 μM) and *N*-acetylcysteine (positive control). After incubation for 2 h, the cells were further incubated for 24 h in the presence of 50 μM STZ. Then, INS-1 cells were washed twice with Hank's Balanced Salt Solution (HBSS) and incubated with DCF-DA (2.5 μM) in the dark for 10 min, followed by another round of washing with HBSS to remove DCF-DA. The DCF fluorescence was measured using a fluorescence plate reader (Tecan Infinite F200 microplate fluorescence reader, Männedorf, Switzerland) at excitation (485 nm) and emission (530 nm) wavelengths. 

### 4.5. Insulin Secretion Assay

To determine the level of GSIS, INS-1 cells were seeded into a 12-well cell culture plate at a density of 5 × 10^5^ cells/mL (500 μL/well) [43]. After 24 h, the medium was washed twice with Krebs-Ringer bicarbonate HEPES buffer (KRBB, 4.8 mM KCl, 129 mM NaCl, 1.2 mM KH_2_PO_4_, 1.2 mM MgSO_4_, 2.5 mM CaCl_2_, 10 mM HEPES, 5 mM NaHCO_3_, and 0.1% bovine serum albumin (BSA), pH 7.4) and 2.8 mM glucose. Cells were allowed to starve in new KRBB for 2 h. Then the buffer was removed and the cells were treated with α-mangostin (1, 2.5, 5 μM) and gliclazide (positive control). Next, low and high glucose concentrations (3.3 mM and 16.7 mM, respectively) were added to the culture medium. Following a further incubation period of 1 h, insulin concentration was determined via ELISA using the rat insulin ELISA kit according to the instructions. Supernatants of cells were centrifuged at 12,000 rpm and 4 °C for 10 min and then collected to measure the concentration of insulin. GSIS was calculated as the ratio between the insulin concentration after stimulation by high glucose concentration and insulin concentration after stimulation by low glucose concentration. A fold stimulation of insulin secretion was calculated by dividing the mean GSIS in high glucose concentration (16.7 mM) by the mean GSIS in low glucose concentrations (3.3 mM glucose). The values were normalized to total protein amount from cell lysates, determined using the Pierce™ BCA protein assay kit (Thermo Fisher Scientific, Waltham, MA, USA). 

### 4.6. Western Blot Analysis

The cellular pathway was determined by Western blotting analysis [44]. Conditioned INS-1 cells were collected and lysed with RIPA buffer containing 1 mM phenylmethylsulfonyl fluoride on ice. The concentration of each protein was determined using the Pierce™ BCA protein assay kit. The proteins (20 μg/lane) were electrophoresed in 10% sodium dodecyl sulfate polyacrylamide gel, blotted onto polyvinylidene difluoride membranes, and further incubated for 1 h with primary antibodies against phospho-insulin receptor (P-IR), phospho-insulin receptor substrate-1 (P-IRS-1 (Ser1101)), phospho-phosphatidylinositol-3 kinase (P-PI3K), phospho-Akt (P-Akt), phospho-ERK (P-ERK), pancreatic and duodenal homeobox 1 (Pdx1), phospho-p38 (P-p38), p38, phospho-c-Jun N-terminal kinase (P-JNK), JNK, cleaved caspase-3, and glyceraldehyde 3-phosphate dehydrogenase (GAPDH) (Cell Signaling, Boston, MA, USA) at room temperature. Following incubation with horseradish peroxidase (HRP)-conjugated anti-rabbit secondary antibodies (Cell Signaling, Boston, MA, USA) for 1 h at room temperature, the proteins were visualized using the chemiluminescence system (FUSION Solo, PEQLAB Biotechnologie GmbH, Erlangen, Germany) in the presence of Western blotting detection reagents (ECL Advance, GE Healthcare, Cambridge, UK) according to the instructions.

### 4.7. Staining for Intracellular Ca^2+^

To determine the level of intracellular Ca^2+^, INS-1 cells were seeded into a 12-well cell culture plate at a density of 5 × 10^5^ cells/mL (500 μL/well). After 24 h, the medium was washed twice with Krebs-Ringer bicarbonate HEPES buffer (KRBB, 4.8 mM KCl, 129 mM NaCl, 1.2 mM KH_2_PO_4_, 1.2 mM MgSO_4_, 2.5 mM CaCl_2_, 10 mM HEPES, 5 mM NaHCO_3_, and 0.1% BSA, pH 7.4) and 2.8 mM glucose. Cells were allowed to starve in new KRBB for 2 h. Then the buffer was removed and the cells were treated with α-mangostin (5 μM). Next, high glucose concentrations (16.7 mM) were added to the culture medium. After 1 h, 24 h, and 48 h, cells were stained with 2 µM Fluo-4 AM (Invitrogen, Eugene, OR, USA), a fluorescent indicator for Ca^2+^. Fluorescent Ca^2+^ images were captured digitally using a fluorescent microscope equipped with a cooled CCD camera (Olympus, Tokyo, Japan).

### 4.8. Statistical Analysis

All data including cell viability, glucose stimulation index, fluorescence intensity, and protein expressions were presented as the average value and standard deviation (SD). All the assays were done in triplicate for each assay and were repeated at least three times. In this study, only a few repetitions of each of the cell experiments were included, thus the non-parametric analysis method was adopted for statistical analysis. The Kruskall-Wallis test was used for the statistical analysis of each variable. SPSS statistical package was used for all analyses (IBM SPSS statistics version 21, Boston, MA, USA). Statistical significance was considered at a *p*-value lower than 0.05.

## 5. Conclusions

In the present study, our results showed that α-mangostin stimulated insulin secretion in INS-1 cells via activating IR and Pdx1 followed by PI3K, Akt, and ERK signaling cascades, whereas it inhibited the phosphorylation of IRS-1 (Ser1101). Moreover, α-mangostin was found to restore the STZ-induced decrease in intracellular ROS levels and INS-1 cell viability in a dose-dependent manner. Similarly, STZ caused a marked increase in the phosphorylation of P38, JNK, and cleaved caspase-3, which was decreased significantly by co-treatment with 5 μM α-mangostin. This study demonstrates that α-mangostin is capable of improving insulin secretion in pancreatic β-cells and protecting cells from apoptotic damage.

## Supplementary Materials

Supplementary materials can be found at http://www.mdpi.com/1422-0067/19/5/1484/s1.
