# Supplementary material for: Alpha-Mangostin Improves Insulin Secretion and Protects INS-1 Cells from Streptozotocin-Induced Damage
Source: Int J Mol Sci. 2018 May 16;19(5):1484. doi: 10.3390/ijms19051484 (PMC5983655; doi:10.3390/ijms19051484)
Supplement: Supplementary file 1 [file ijms-19-01484-s001.zip › ijms-294271-SI.pdf]

Supporting Information

Alpha-mangostin improves insulin secretion and protects INS-1 cells from streptozotocin-induced damage

Dahae Lee[1,2,†], Young-Mi Kim[3,†], Kiwon Jung[4,†], Young-Won Chin[3,*], Ki Sung Kang[2,*]

[1] School of Pharmacy, Sungkyunkwan University, Suwon 16419, Republic of Korea; pjsldh@naver.com
[2] College of Korean Medicine, Gachon University, Seongnam 13120, Republic of Korea; kkang@gachon.ac.kr
[3] College of Pharmacy and Integrated Research Institute for Drug Development, Dongguk University-Seoul, Gyeonggi 10326, Korea; 0210121@hanmail.net; f2744@dongguk.edu
[4] Institute of Pharmaceutical Sciences, College of Pharmacy, CHA University, Sungnam 13844, Korea; pharmj@cha.ac.kr
[†] These authors contributed equally to this work
**Correspondence: f2744@dongguk.edu; Tel.: + 82-31-961-5218 (Y.W.C.) and kkang@gachon.ac.kr; Tel.: + 82-31-750-5402 (K.S.K.)*

Contents

Figure S1. The ^{1}H NMR spectrum of α-mangostin (CD_3OD, 400 MHz) 2

Figure S2. The ^{13}C NMR spectrum of α-mangostin (CD_3OD, 100 MHz) 3

Figure S3. The UPLC UV chromatogram of α-mangostin (detected at 320 nm) 4

Figure S4. The MASS spectra of α-mangostin 5

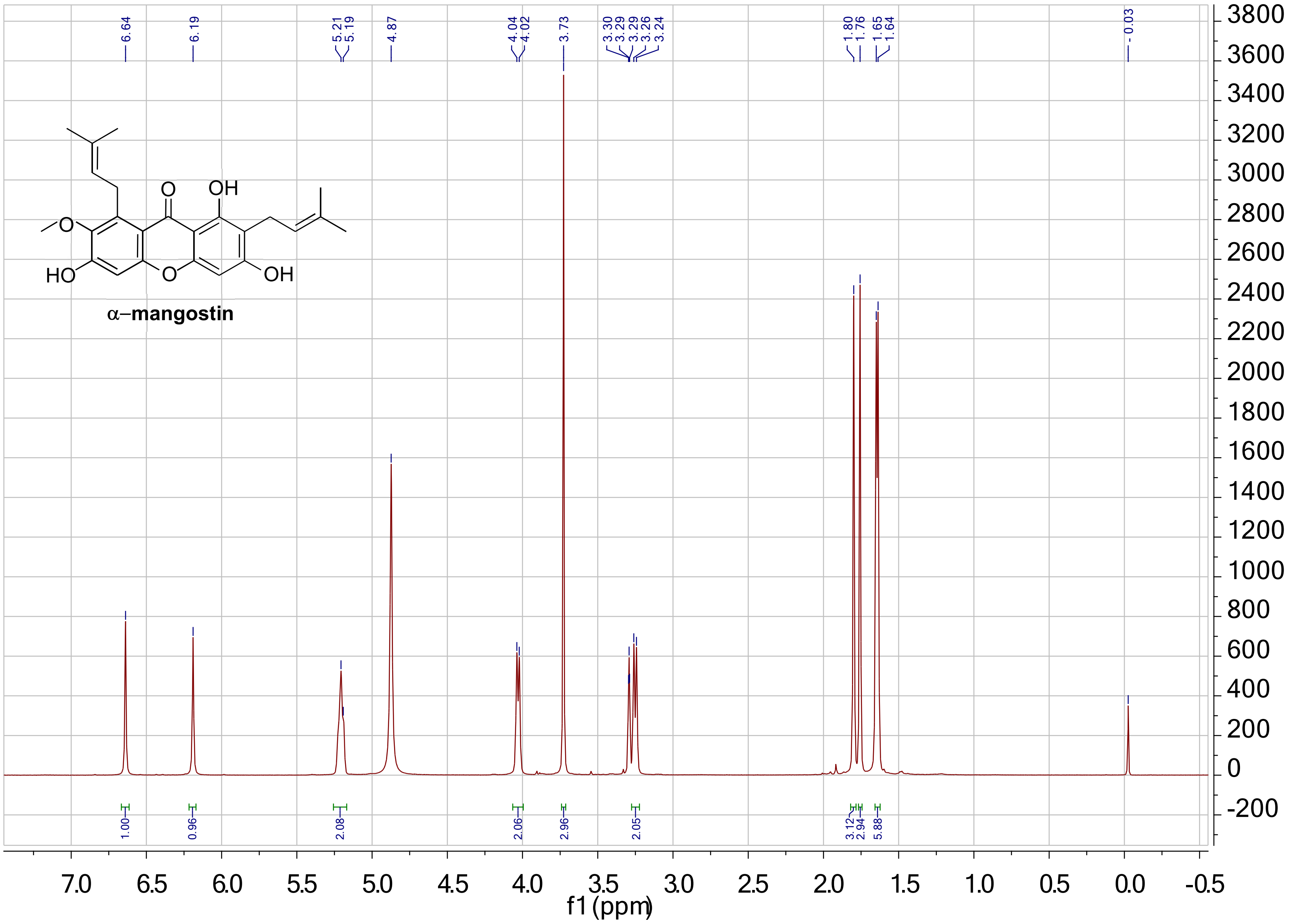

Figure S1. The ^{1}H NMR spectrum of α-mangostin in (CD_3OD, 400 MHz)

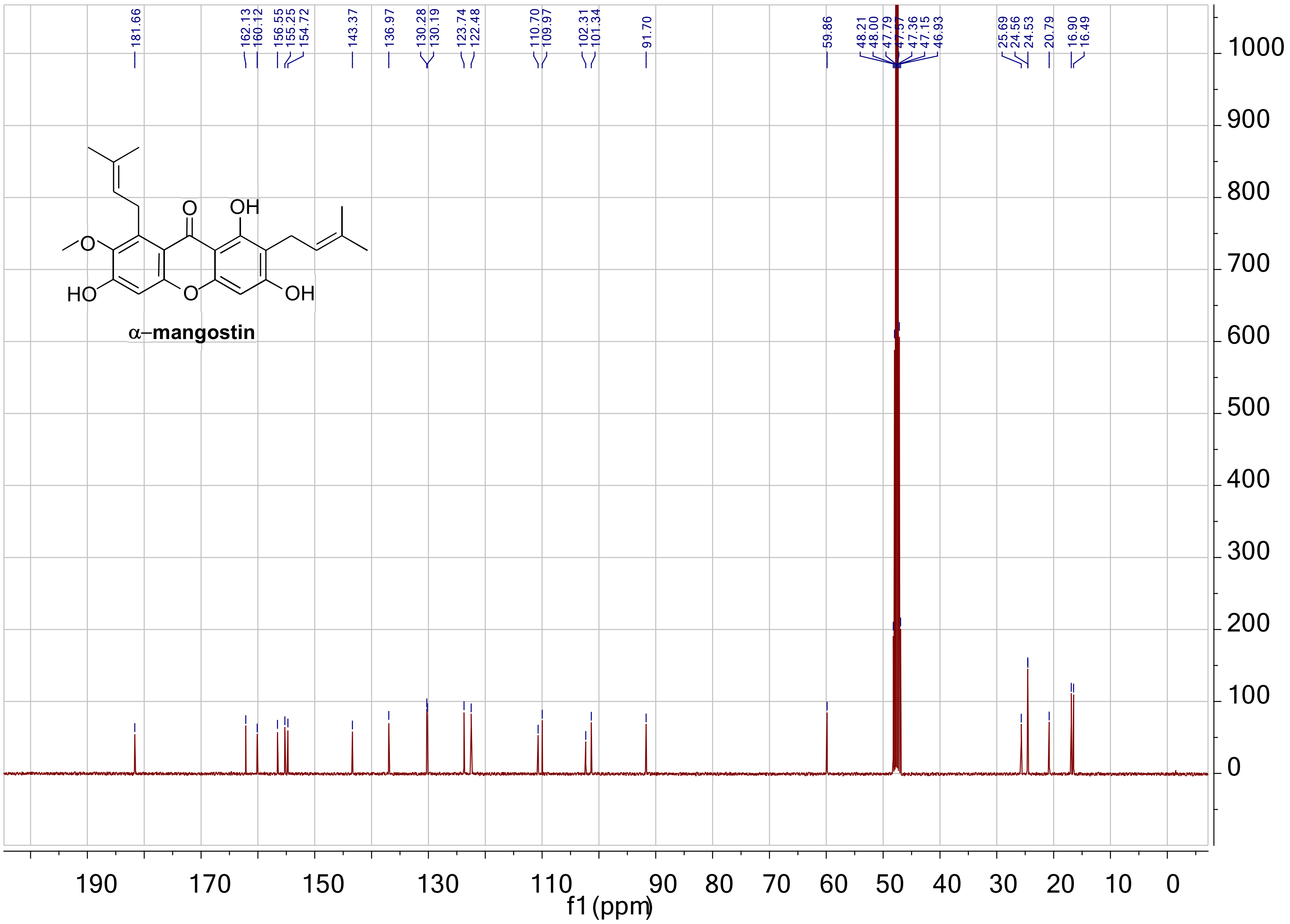

Figure S2. The ^{13}C NMR spectrum of α-mangostin (CD_3OD, 100 MHz)

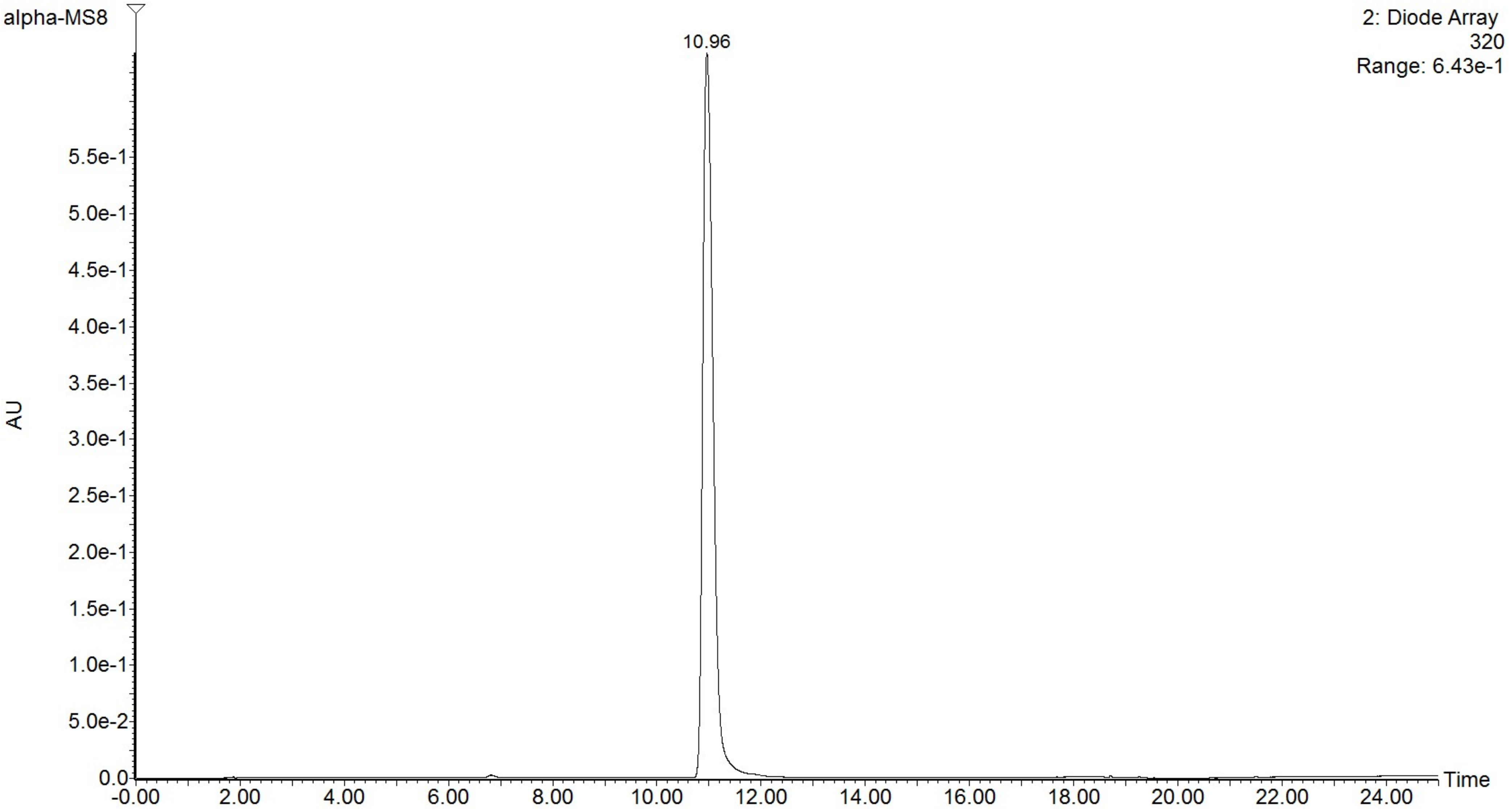

Figure S3. The UPLC UV chromatogram of α-mangostin (UV wavelength 320nm)

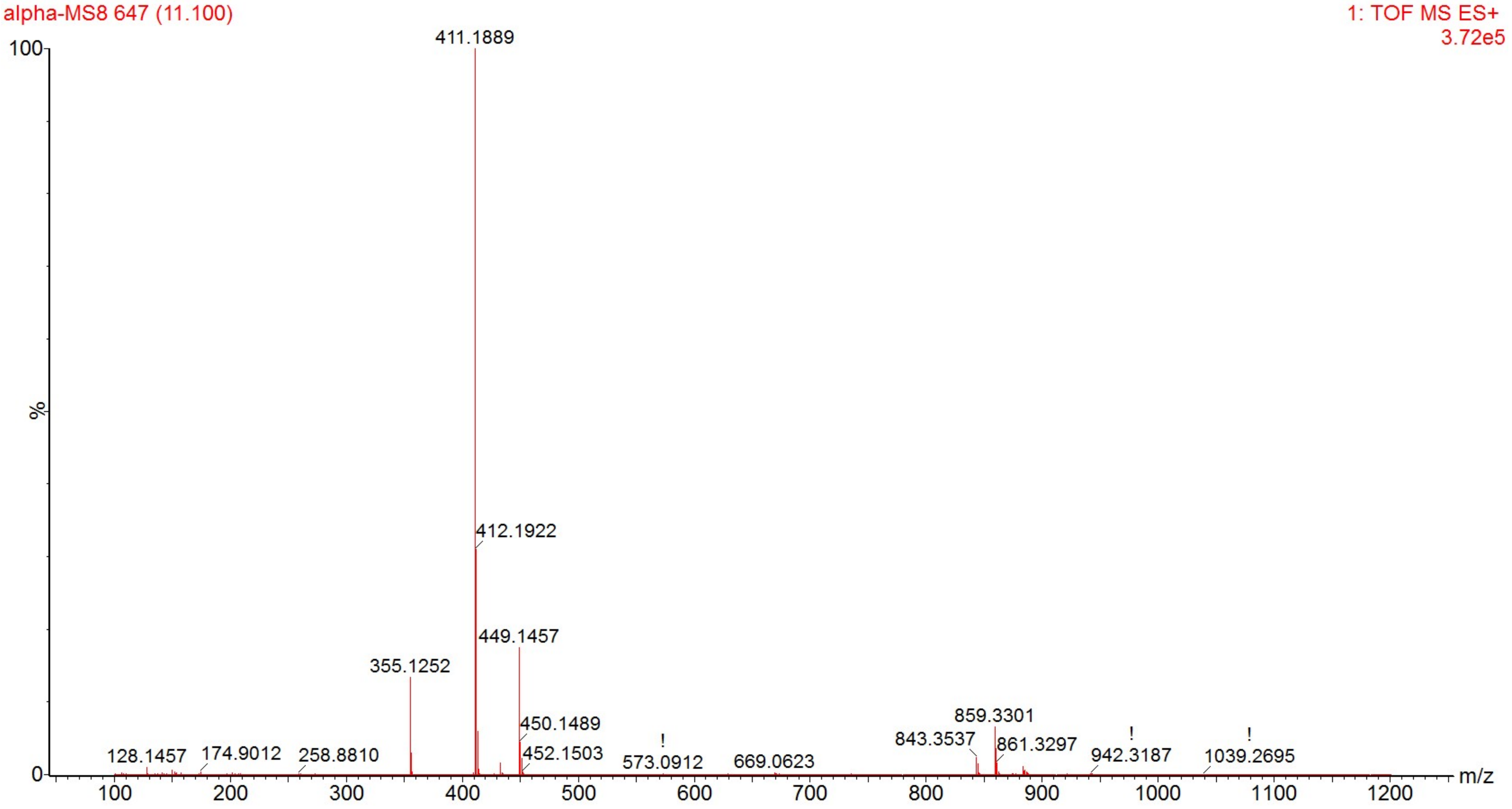

Figure S4. The MASS spectra of α-mangostin
